# Gene model for the ortholog of *Pten* in *Drosophila ananassae*

**DOI:** 10.17912/micropub.biology.000901

**Published:** 2025-08-12

**Authors:** Graham M. Jones, D'Andrew Harrington, Zachary Lill, Amy T. Hark, Chelsey McKenna, Chinmay P. Rele, Laura K Reed

**Affiliations:** 1 University of Alabama, Tuscaloosa, AL USA; 2 College of Southern Nevada, Henderson, NV USA; 3 Muhlenberg College, Allentown, PA USA

## Abstract

Gene model for the ortholog of *Phosphatase and tensin homolog* ( *Pten* ) in the May 2011 (Agencourt dana_caf1/DanaCAF1) Genome Assembly (GenBank Accession: GCA_000005115.1 ) of *Drosophila ananassae* . This ortholog was characterized as part of a developing dataset to study the evolution of the Insulin/insulin-like growth factor signaling pathway (IIS) across the genus *Drosophila* using the Genomics Education Partnership gene annotation protocol for Course-based Undergraduate Research Experiences.

**Figure 1. Genomic neighborhood and gene model for Pten in Drosophila ananassae:**
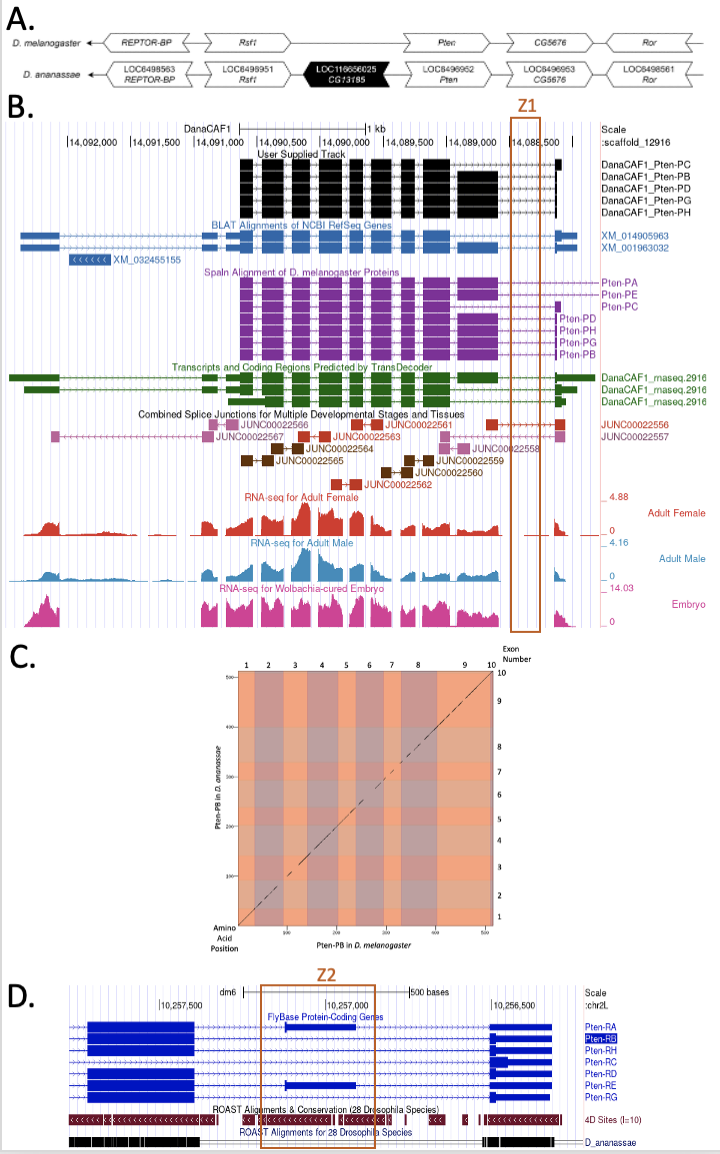
**
(A) Synteny comparison of the genomic neighborhoods for *Pten* in *Drosophila melanogaster* and *D. ananassae* .** The thin arrows pointing to the left indicate that *Pten* is on the negative (-) strand in *D. melanogaster* (top) and *D. ananassae* (bottom). The wide gene arrows pointing in the same direction as *Pten* are on the same strand relative to the thin underlying arrows, while wide gene arrows pointing in the opposite direction of *Pten* are on the opposite strand relative to the thin underlying arrows. White gene arrows in *D. ananassae* indicate orthology to the corresponding gene in *D. melanogaster* , while black gene arrows indicate non-orthology. Gene symbols given in the *D. ananassae* gene arrows indicate the orthologous gene in *D. melanogaster* , while the locus identifiers are specific to *D. ananassae* . **(B) Gene Model in GEP UCSC Track Data Hub** (Raney et al., 2014). The coding-regions of *Pten* in *D. ananassae* are displayed in the User Supplied Track (black); CDSs are depicted by thick rectangles and introns by thin lines with arrows indicating the direction of transcription. Subsequent evidence tracks include BLAT Alignments of NCBI RefSeq Genes (dark blue, alignment of Ref-Seq genes for *D. ananassae* ), Spaln of *D. melanogaster* Proteins (purple, alignment of Ref-Seq proteins from *D. melanogaster* ), Transcripts and Coding Regions Predicted by TransDecoder (dark green), RNA-Seq from Adult Females, Adult Males and Wolbachia-cured Embryos (red, light blue and pink, respectively); Splice Junctions Predicted by regtools using *D. ananassae* RNA-Seq, and alignment of Illumina RNA-Seq reads from *D. ananassae* ( SRP006203 , SRP007906 ; PRJNA257286 , PRJNA388952 ). Splice junctions shown have a read-depth of 100-499, 500-999, >1000 supporting reads in pink, brown, and red, respectively. The region enclosed in orange, denoted Z1, highlights the region between the ninth and tenth CDS of *Rten-RB* (and all identical isoforms). **(C) Dot Plot of Pten-PB in *D. melanogaster* ( *x* -axis) vs. the orthologous peptide in *D. ananassae* ( *y* -axis).** Amino acid number is indicated along the left and bottom; CDS number is indicated along the top and right, and CDSs are also highlighted with alternating colors. Line breaks in the dot plot indicate mismatching amino acids at the specified location between species. **(D) Ninth and tenth CDSs *Pten* isoforms displayed in *D. melanogaster* UCSC Genome Browser with ROAST Alignments of 28 Drosophila Species track exhibiting conservation of sequence in *D. ananassae* .** Evidence tracks include FlyBase Protein-Coding Genes of *Pten* isoforms in *D. melanogaster* (blue), ROAST Alignments and Conservation (28 Drosophila Species) (burgundy) and conservation of sequence in *D. ananassae* (black). The final CDS of *Pten-RA* and *Pten-RE* is highlighted in orange, denoted Z2. The ROAST Alignment track (black) supports the absence of this CDS and its isoform ( *Pten-RA* ; *Pten-RE* ) in *D. ananassae* .

## Description

**Table:** 

*This article reports a predicted gene model generated by undergraduate work using a structured gene model annotation protocol defined by the Genomics Education Partnership (GEP; thegep.org) for Course-based Undergraduate Research Experience (CURE). The following information in this box may be repeated in other articles submitted by participants using the same GEP CURE protocol for annotating Drosophila species orthologs of Drosophila melanogaster genes in the insulin signaling pathway.* "In this GEP CURE protocol students use web-based tools to manually annotate genes in non-model *Drosophila* species based on orthology to genes in the well-annotated model organism fruitfly *Drosophila melanogaster* . The GEP uses web-based tools to allow undergraduates to participate in course-based research by generating manual annotations of genes in non-model species (Rele et al., 2023). Computational-based gene predictions in any organism are often improved by careful manual annotation and curation, allowing for more accurate analyses of gene and genome evolution (Mudge and Harrow 2016; Tello-Ruiz et al., 2019). These models of orthologous genes across species, such as the one presented here, then provide a reliable basis for further evolutionary genomic analyses when made available to the scientific community.” (Myers et al., 2024). “The particular gene ortholog described here was characterized as part of a developing dataset to study the evolution of the Insulin/insulin-like growth factor signaling pathway (IIS) across the genus *Drosophila* . The Insulin/insulin-like growth factor signaling pathway (IIS) is a highly conserved signaling pathway in animals and is central to mediating organismal responses to nutrients (Hietakangas and Cohen 2009; Grewal 2009).” (Myers et al., 2024). “The *Drosophila Phosphatase and tensin homolog* ( *Pten* also known as *dPTEN* ; FBgn0026379), identified due to is conservation to the human tumor suppressor gene acts as a protein and lipid phosphatase in the insulin signaling pathway (Goberdhan et al., 1999; Huang et al., 1999). Pten is known to affect cell number and size through the inhibition of the phosphoinositide 3-kinase (PI3K) and AKT kinase pathways (Goberdhan et al., 1999; Gao et al., 2000). Pten is involved in stabilizing cell junctions (Bardet et al., 2013) and regulates the cytoskeleton, controlling the localization and organization of actin (Goberdhan et al., 1999; von Stein et al., 2005).” (Lawson et al., 2024a). “ *D* . *ananassae* (NCBI:txid7217) is part of the *melanogaster* species group within the subgenus *Sophophora* of the genus *Drosophila* (Sturtevant 1939; Bock and Wheeler 1972). It was first described by Doleschall (1858). *D. ananassae* is circumtropical (Markow and O'Grady 2005; https://www.taxodros.uzh.ch, accessed 1 Feb 2023), and often associated with human settlement (Singh 2010). It has been extensively studied as a model for its cytogenetic and genetic characteristics, and in experimental evolution (Kikkawa 1938; Singh and Yadav 2015).” (Lawson et al., 2024b).

We propose a gene model for the *D. ananassae* ortholog of the *D. melanogaster* *Phosphatase and tensin homolog* ( *Pten* ) gene. The genomic region of the ortholog corresponds to the uncharacterized protein XP_001963068.1 (Locus ID LOC6496952 ) in the *D. ananassae* May 2011 (Agencourt dana_caf1/DanaCAF1) Genome Assembly of *D. ananassae* ( GCA_000005115.1 - *Drosophila* 12 Genomes Consortium et al., 2007). This model is based on RNA-Seq data from *D. ananassae* ( SRP006203 , SRP007906 ; PRJNA257286 , PRJNA388952 - Graveley et al., 2011) and *Pten* in *D. melanogaster* using FlyBase release FB2023_03 ( GCA_000001215.4 ; Larkin et al., 2021; Gramates et al., 2022; Jenkins et al., 2022).

***Synteny***

The target gene, *Pten ,* occurs on chromosome 2L in *D. melanogaster* and is flanked upstream by *REPTOR-binding partner* ( *REPTOR-BP* ) and *Repressor splicing factor 1* ( *Rsf1* ) and downstream by *CG5676* and *Ror* ( *Ror* ). The *tblastn* search of *D. melanogaster* Pten-PB (query) against the *D. ananassae* (GenBank Accession: GCA_000005115.1 ) Genome Assembly (database) placed the putative ortholog of *Pten* within scaffold scaffold_12916 ( CH902620.1 ) at locus LOC6496952 ( XP_001963068.1 ), with an E-value of 2e-82 and a percent identity of 92.78%. Furthermore, the putative ortholog is flanked upstream by LOC6498563 ( XP_001963071.2 ), LOC6496951 ( XP_032311424.1 ) and LOC116656025 ( XP_032311046.1 ), which correspond to *REPTOR-BP* , *Rsf1* and *CG13185* in *D. melanogaster* (E-value: 1e-67, 2e-80 and 6e-32; identity: 83.90%, 82.50% and 67.39%, respectively, as determined by *blastp* ; Figure 1A ; Altschul et al., 1990). The putative ortholog of *Pten* is flanked downstream by LOC6496953 ( XP_001963067.1 ) and XP_001963067.1 ( XP_001963066.2 ), which correspond to *CG5676* and *Ror* in *D. melanogaster* (E-value: 1e-104 and 0.0; identity: 91.67% and 93.00%, respectively, as determined by *blastp* ). The putative ortholog assignment for *Pten* in *D. ananassae* is supported by the following evidence: The genes surrounding the *Pten* ortholog are orthologous to the genes at the same locus in *D. melanogaster* and local synteny is nearly completely conserved (aside from the insertion of *CG13185* ), supported by results generated from *blastp* , so we conclude that LOC6496952 is the correct ortholog of *Pten* in *D. ananassae* ( Figure 1A ).

***Protein Model***

*Pten* in *D. ananassae* has two unique protein-coding isoforms: Pten-PB (identical to Pten-PH, Pten-PG and Pten-PD) and Pten-PC ( Figure 1B, black). mRNA isoforms *Pten-RB* , *Pten-RH* , *Pten-RG* and *Pten-RD* contain ten CDSs and mRNA isoform *Pten-RC* contains nine CDSs. The two isoforms differ due to alternative splice junctions JUNC 00022557 and 00022558. Relative to the ortholog in *D. melanogaster* , the CDS number and protein isoform count are not-conserved. In *D. melanogaster, Pten-RB* ( *Pten-RH* , *Pten-RG* and *Pten-RD* ) contains nine CDSs and *Pten-RC* contains eight CDSs. In addition, *Pten* in *D. melanogaster* contains an additional unique isoform, *Pten-RA* (identical to *Pten-RE* ), which contains ten CDSs. The sequence of Pten-PB in *D. ananassae* has 82.72% identity (E-value: 0.0) with the protein-coding isoform Pten-PB in *D. melanogaster* , as determined by *blastp* ( Figure 1C ). Coordinates of this curated gene model are stored by NCBI at GenBank/BankIt (accession BK064655 , BK064656 , BK064657 , BK064658 , BK064659 ). These data are also archived in the CaltechDATA repository (see “Extended Data” section below).

***Special characteristics of the protein model***

**Putative absence of *Pten-RA* and *Pten-RE* in *D. ananassae* :** *D. melanogaster* contains three unique protein isoforms: Pten-PB (Pten-PH, Pten-PG and Pten-PD), Pten-PC, and Pten-PA (Pten-PE). Only two of these unique isoforms have been confirmed in *D. ananassae* : Pten-PB (Pten-PH, Pten-PG and Pten-PD) and Pten-PC ( Figure 1B, black). In *D. melanogaster* , *Pten-RA* ( *Pten-RE* ) uses an alternative splice acceptor site for its final CDS, located between the ninth and tenth CDS of *Pten-RB* (and identical isoforms). Lack of RNA-Seq expression and predicted splice junctions in the expected region of the final CDS of *Pten-RA* ( *Pten-RE* ), highlighted in orange (Z1), support the absence of this unique isoform in *D. ananassae* ( Figure 1B ). Furthermore, conservation of sequence of *D. ananassae* by ROAST Alignments and Conservation (28 Drosophila Species) displays a lack of conservation in the region of the expected final CDS of *Pten-RA* ( *Pten-RE* ), indicating the loss of Pten-PA and Pten-PE in *D. ananassae* ( Figure 1D, black).

## Methods

Detailed methods including algorithms, database versions, and citations for the complete annotation process can be found in Rele et al. (2023). Briefly, students use the GEP instance of the UCSC Genome Browser v.435 (https://gander.wustl.edu; Kent WJ et al., 2002; Navarro Gonzalez et al., 2021) to examine the genomic neighborhood of their reference IIS gene in the *D. melanogaster* genome assembly (Aug. 2014; BDGP Release 6 + ISO1 MT/dm6). Students then retrieve the protein sequence for the *D. melanogaster* reference gene for a given isoform and run it using *tblastn* against their target *Drosophila* species genome assembly on the NCBI BLAST server (https://blast.ncbi.nlm.nih.gov/Blast.cgi; Altschul et al., 1990) to identify potential orthologs. To validate the potential ortholog, students compare the local genomic neighborhood of their potential ortholog with the genomic neighborhood of their reference gene in *D. melanogaster* . This local synteny analysis includes at minimum the two upstream and downstream genes relative to their putative ortholog. They also explore other sets of genomic evidence using multiple alignment tracks in the Genome Browser, including BLAT alignments of RefSeq Genes, Spaln alignment of *D. melanogaster* proteins, multiple gene prediction tracks (e.g., GeMoMa, Geneid, Augustus), and modENCODE RNA-Seq from the target species. Detailed explanation of how these lines of genomic evidenced are leveraged by students in gene model development are described in Rele et al. (2023). Genomic structure information (e.g., CDSs, intron-exon number and boundaries, number of isoforms) for the *D. melanogaster* reference gene is retrieved through the Gene Record Finder (https://gander.wustl.edu/~wilson/dmelgenerecord/index.html; Rele et al *.,* 2023). Approximate splice sites within the target gene are determined using *tblastn* using the CDSs from the *D. melanogaste* r reference gene. Coordinates of CDSs are then refined by examining aligned modENCODE RNA-Seq data, and by applying paradigms of molecular biology such as identifying canonical splice site sequences and ensuring the maintenance of an open reading frame across hypothesized splice sites. Students then confirm the biological validity of their target gene model using the Gene Model Checker (https://gander.wustl.edu/~wilson/dmelgenerecord/index.html; Rele et al., 2023), which compares the structure and translated sequence from their hypothesized target gene model against the *D. melanogaster* reference gene model. At least two independent models for a gene are generated by students under mentorship of their faculty course instructors. Those models are then reconciled by a third independent researcher mentored by the project leaders to produce the final model. Note: comparison of 5' and 3' UTR sequence information is not included in this GEP CURE protocol (Gruys et al., 2025).

## Data Availability

Description: GFF, FASTA, and PEP of Pten in Drosophila ananassae. Resource Type: Model. DOI: https://doi.org/10.22002/p0pst-n6h15
